# Controllable crystal form transformation and luminescence properties of up-conversion luminescent material K_3_Sc_0.5_Lu_0.5_F_6_: Er^3+^, Yb^3+^ with cryolite structure

**DOI:** 10.1039/d1ra06258a

**Published:** 2021-09-08

**Authors:** Zhaoliang Yan, Qingfeng Guo, Libing Liao, Pengfei Shuai, Feifei Huang, Lefu Mei

**Affiliations:** Beijing Key Laboratory of Materials Utilization of Nonmetallic Minerals and Solid Wastes, National Laboratory of Mineral Materials. School of Materials Sciences and Technology, China University of Geosciences Beijing 100083 China clayl@cugb.edu.cn; School of Gemology, China University of Geosciences, Jewelry and Mineral Materials Laboratory of Experimental Teaching Demonstration Center Beijing 100083 China

## Abstract

In this paper, a novel cryolite-type up-conversion luminescent material K_3_Sc_0.5_Lu_0.5_F_6_: Er^3+^, Yb^3+^ with controllable crystal form was synthesized by a high temperature solid state method. K_3_Sc_0.5_Lu_0.5_F_6_: Er^3+^, Yb^3+^ can crystallize in monoclinic or cubic form at different temperatures. The composition, structure and up-conversion luminescence (UCL) properties of K_3_Sc_0.5_Lu_0.5_F_6_: Er^3+^, Yb^3+^ samples with different crystal form were investigated in detail. It is impressive that both monoclinic and cubic forms of K_3_Sc_0.5_Lu_0.5_F_6_: Er^3+^, Yb^3+^ show green emission (^2^H_11/2_/^4^S_3/2_→^4^I_15/2_). The luminescence intensity of cubic K_3_Sc_0.5_Lu_0.5_F_6_ is much higher than that of the monoclinic form, and the reasons are also discussed in detail. The results show that the luminescence intensity of up-conversion materials can be effectively tuned by controlling the crystal form. According to the power dependent UCL intensity, the UCL mechanism and electronic transition process were discussed. In addition, the fluorescence decay curves were characterized and the thermal coupling levels (TCLs) of Er^3+^ (^2^H_11/2_/^4^S_3/2_ → ^4^I_15/2_) in the range of 304–574 *k* were used to study the optical temperature sensing characteristics. All the results show that K_3_Sc_0.5_Lu_0.5_F_6_: Er^3+^, Yb^3+^ can be used in electronic components and have potential application value in temperature sensing fields.

## Abstract 2

Controllable crystal form transformation can effectively influence the up-conversion luminous intensity of cryolite materials.

## Graphical abstract

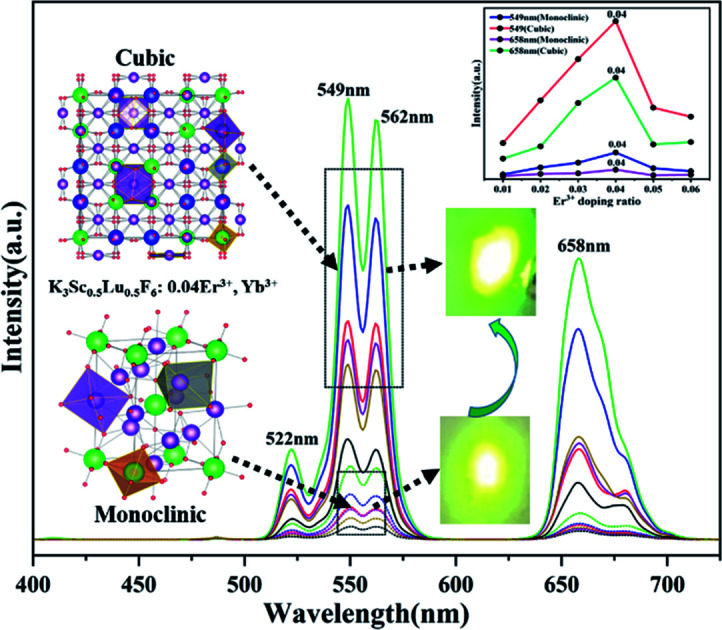

## 1. Introduction

Lanthanides are usually used as the luminescence center of up-conversion luminescent materials due to their abundant energy levels, efficient energy conversion, and unique optical properties.^1,2^ Lanthanides can be excited by an external light source and the electrons of lanthanides can jump between different energy levels, showing the absorption of photons and up-conversion luminescence characteristics. Lanthanides have different electron configuration and energy level structure, therefore exhibit different up-conversion luminescence properties.^3–8^ Er^3+^/Yb^3+^ pairs are the most attractive lanthanide ions for up-conversion luminescence. Er^3+^ can absorb near-infrared photons around 980 nm and emit green and red light through the up-conversion process. Therefore, Er^3+^ is considered to be a superior up-conversion luminous center. However, Er^3+^ has weak absorption in the visible and near-infrared region, and it needs to be sensitized by other ions. Yb^3+^ is the most effective sensitizing ion because it has a large absorption cross-section and a wide absorption region and the energy level of Yb^3+^ and Er^3+^ matches well. Therefore, there is an effective energy transfer between Er^3+^ and Yb^3+^, and the up-conversion luminous efficiency of Er^3+^ is significantly improved.^9–15^

The most common way to improve the efficiency of up-conversion is to use a host with low phonon energy.^16^ We hope to find materials with suitable crystal field environment and higher temperature sensitivity around Er^3+^, which can further improve the performance of optical temperature sensor. The fluoride host has significant physical and chemical properties and low phonon energy, which makes it suitable for non-contact optical temperature measurement. Many previous works have reported the application of fluoride in temperature sensing. Baziulyte-Paulaviciene successfully synthesized Er^3+^ doped hexagonal NaYbF_4_ particles, which can work in the temperature range of 175–475 K, and reach the maximum relative sensor sensitivity of 3.46% K^−1^ at 175 K.^17^ Qiang synthesized Mn^2+^ co-doped hexagonal NaGdF_4_: Yb^3+^, Ho^3+^ nano-phosphor, and proved that high-concentration Mn^2+^ doping can improve the sensing sensitivity of the sample.^18^ Kumar successfully doped GdF_3_: Ho^3+^, Yb^3+^ phosphors with Ag^+^ to achieve emission enhancement and real-time temperature sensing through magnetic field adjustment.^19^ Besides, Du synthesized SrF_2_: Yb^3+^, Ho^3+^ and realized wide-range temperature sensing.^20^

Cryolite is a promising host material in the field of luminescent materials due to its low phonon energy, good optical transparency, high mechanical and chemical stability.^21,22^ The general structural formula of cryolite is A_3_BF_6_ (A = Li^+^, Na^+^, K^+^, NH^4+^, *etc.* B = Al^3+^, Sc^3+^, Ga^3+^, In^3+^, *etc.*),^23^ and up-conversion luminescent ion pairs can occupy the B site easily, forming a coupled isomorphic replacement in the cryolite crystal lattice. In recent years, research on cryolite structure compound as a luminescent host material has been widely reported, such as K_3_ScF_6_: Tm^3+^, Yb^3+^,^24^ K_3_YF_6_: Er^3+^, Yb^3+ 23^, K_3_LuF_6_: Tb^3+^, Eu^3+^,^25^ K_3_GaF_6_: Mn^4+^,^26^ Na_3_GaF_6_: Eu^3+^,^27^ K_3_GdF_6_,^28^ K_3_AlF_6_: Mn^4+^.^29^ The luminescence characteristics of up-conversion depend on the complex interactions between different doping ions and the host lattice.^30^ Therefore, the luminescence of these particles can be adjusted by the crystal structure of the host lattice, the size of the particles and the ratio of different lanthanide dopants.

Polymorphism refers to the phenomenon that substances with the same chemical composition can crystallize into two or more crystals structures under different physical and chemical conditions. Rare-earth ions in different polymorphic structures will bring about particularly interesting luminescence phenomena. Therefore, the influence of the polymorphism transition on the luminescence properties of rare-earth ions has attracted attention from scholars, such as Gao changed the content ratio of ZnO/Na_2_O in the Tb^3+^–Yb^3+^ co-doped NaYF_4_ nanocrystal-containing zinc fluoride hydroxide glass ceramics to achieve the cubic to hexagonal form transition and the enhancement of up-conversion luminescence;^31^ Janjua prepared ultrafine pure hexagonal NaYF_4_ by the solvothermal method: the up-conversion luminescence intensity of hexagonal NaYF_4_: Er^3+^, Yb^3+^ is 10 times than cubic nanocrystals of the same size.^32^

For cryolite, the most common crystal structures are monoclinic and cubic, and in different crystal form, the up-conversion luminescence properties of cryolite materials maybe different. At present, many cryolite materials with different crystal forms were obtained by substitution of monovalent and trivalent cations, but the comparison of up-conversion luminescence properties of cryolite materials with polymorphism has not been reported. In this article, we synthesized cryolite material: K_3_Sc_0.5_Lu_0.5_F_6_: Er^3+^, Yb^3+^ (KSLF: Er^3+^, Yb^3+^). By changing the synthesis temperature, the monoclinic and cubic KSLF: Er^3+^, Yb^3+^ were obtained. We systematically compared the chemical composition, crystal structure, micromorphology, and up-conversion luminescence properties of KSLF: Er^3+^, Yb^3+^ with different crystal forms. In addition, we also discussed the possible luminescence mechanisms in the two crystal forms, the reasons for the difference in the electronic transition process and the up-conversion luminescence performance, and the application possibility of KSLF: Er^3+^, Yb^3+^ in temperature-sensitive areas.

## 2. Material synthesis and characterization

A series of Er^3+^ and Yb^3+^ co-doped KSLF powders were prepared by high-temperature solid-state method. Potassium carbonate (K_2_CO_3_, A.R.), scandium oxide (Sc_2_O_3_, 99.99), lutetium oxide (Lu_2_O_3_, 99.99), oxidizing bait (Er_2_O_3_, 99.99), ytterbium oxide (Yb_2_O_3_, 99.99), ammonium hydrogen fluoride (NH_4_HF_2_, A.R.) are the raw material, and the above materials are all purchased from Aladdin industrial corporation. Based on the stoichiometric ratio of the target compound, the raw materials are weighed and placed in a mortar and ground for 10 minutes until the mixture is uniformly mixed. Then, the well-mixed ingredients are placed into a crucible and transferred to the muffle furnace. The synthesis temperature of samples with different crystal structures were kept at 800 °C and 900 °C for 3 h and cooling in furnace to room temperature. The obtained samples were ground for subsequent characterization.

X-ray diffraction (XRD) patterns of the synthesized samples were obtained by X-ray powder diffraction (D8 Advance, Bruker, Germany), with the Cu kα = 0.15406 nm, tube current = 40 mA, tube voltage = 40 kV, and the tested 2*θ* range from 10° to 70°, with 0.05° step scan mode. For XRD patterns analysis, the data from JCPDS (Joint Committee on Powder Diffraction Standards) were used as a reference. The size, morphology, energy dispersive X-ray spectroscopy (EDX) and element mapping of the samples were characterized by field emission scanning electron microscopy (SEM, JSM-6701F, Hitachi, Japan), operated at 10 kV. The X-ray photoelectron spectroscopy analyses (XPS, Thermo Escalab 250Xi, American) were performed for elements identification and valence state analysis. TEM images were obtained on a JEM2100F transmission electron microscope. The fluorescence emission spectra of samples were measured on Hitachi F4600 fluorescence spectrophotometer with 980 nm tunable infrared laser as excitation source.

## 3. Results and discussion

**Fig. 1:**
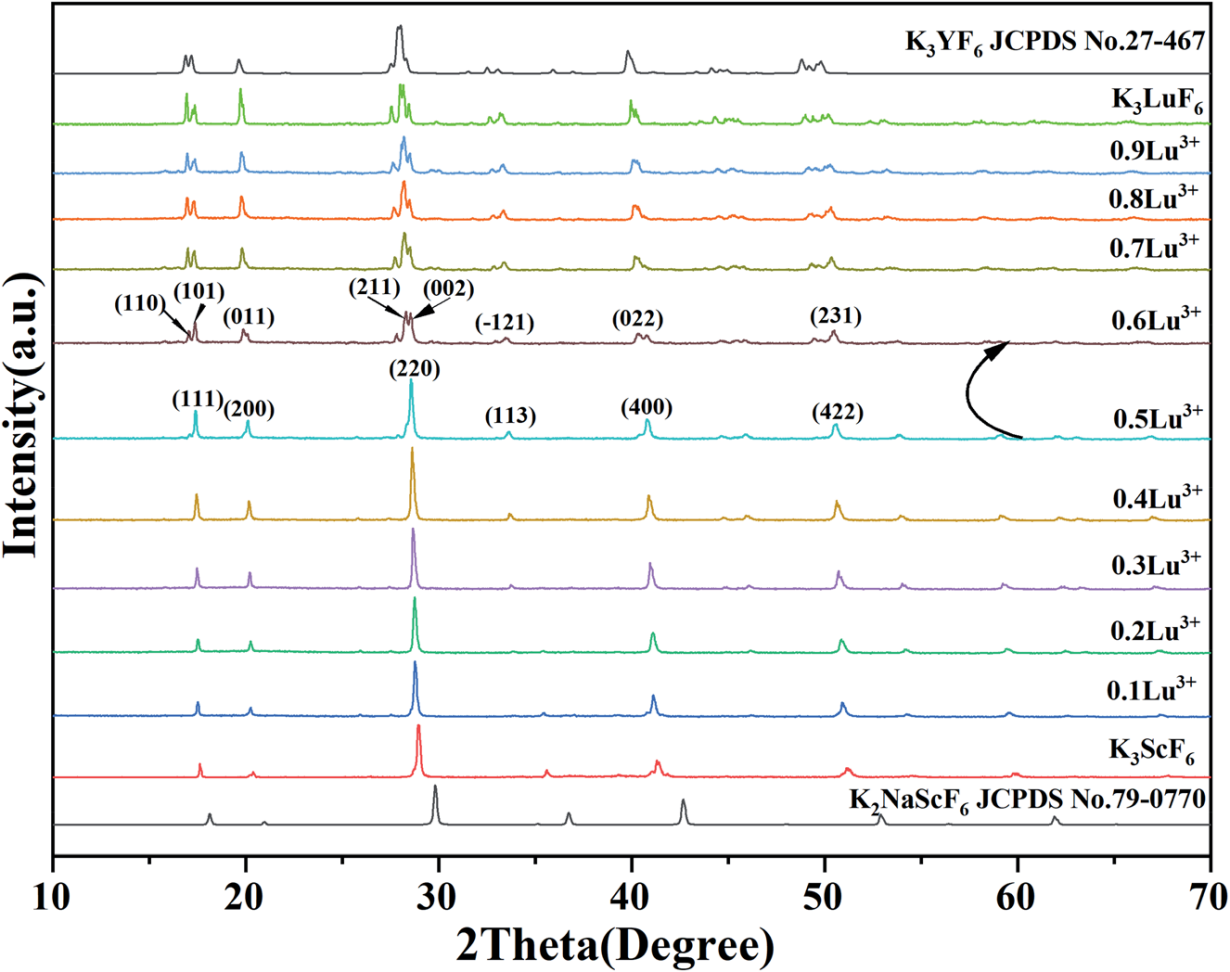
XRD pattern of K_3_Sc_(1−*x*)_Lu_*x*_F_6_ (*x* = 0, 0.1, 0.2, 0.3, 0.4, 0.5, 0.6, 0.7, 0.8, 0.9 and 1) and the standard patterns of K_2_NaScF_6_ (JCPDS no. 79-0770) and K_3_YF_6_ (JCPDF no. 27-467) are shown as reference.

Fig. 1 show the XRD patterns of a series of K_3_Sc_(1−*x*)_Lu_*x*_F_6_ samples synthesized by the high-temperature solid-phase method and the calculated standard profile of cubic K_2_NaScF_6_ (JCPDS no. 79-0770) and monoclinic K_3_YF_6_ (JCPDF no. 27-467) is shown as a reference. All diffraction peaks of the as-prepared samples are in consistent with the standard profile and no other diffraction peaks. When the doping concentration of Lu^3+^ is 50 mol%, the crystal structure of the sample changes from cubic to monoclinic in K_3_Sc_(1−*x*)_Lu_*x*_F_6_. The result shows that replacing Sc^3+^ with Lu^3+^ can effectively affect the transformation of cubic form to monoclinic form.^33,34^ With the increase of Lu^3+^ doping concentration, the XRD diffraction peaks of the sample shift to lower diffraction angles. This can be explain that the Sc^3+^ in the lattice is replaced by a larger Lu^3+^, which leads to the expansion of the unit cell volume, the crystal interplanar spacing (*d*) becomes larger.^35,36^

**Fig. 2:**
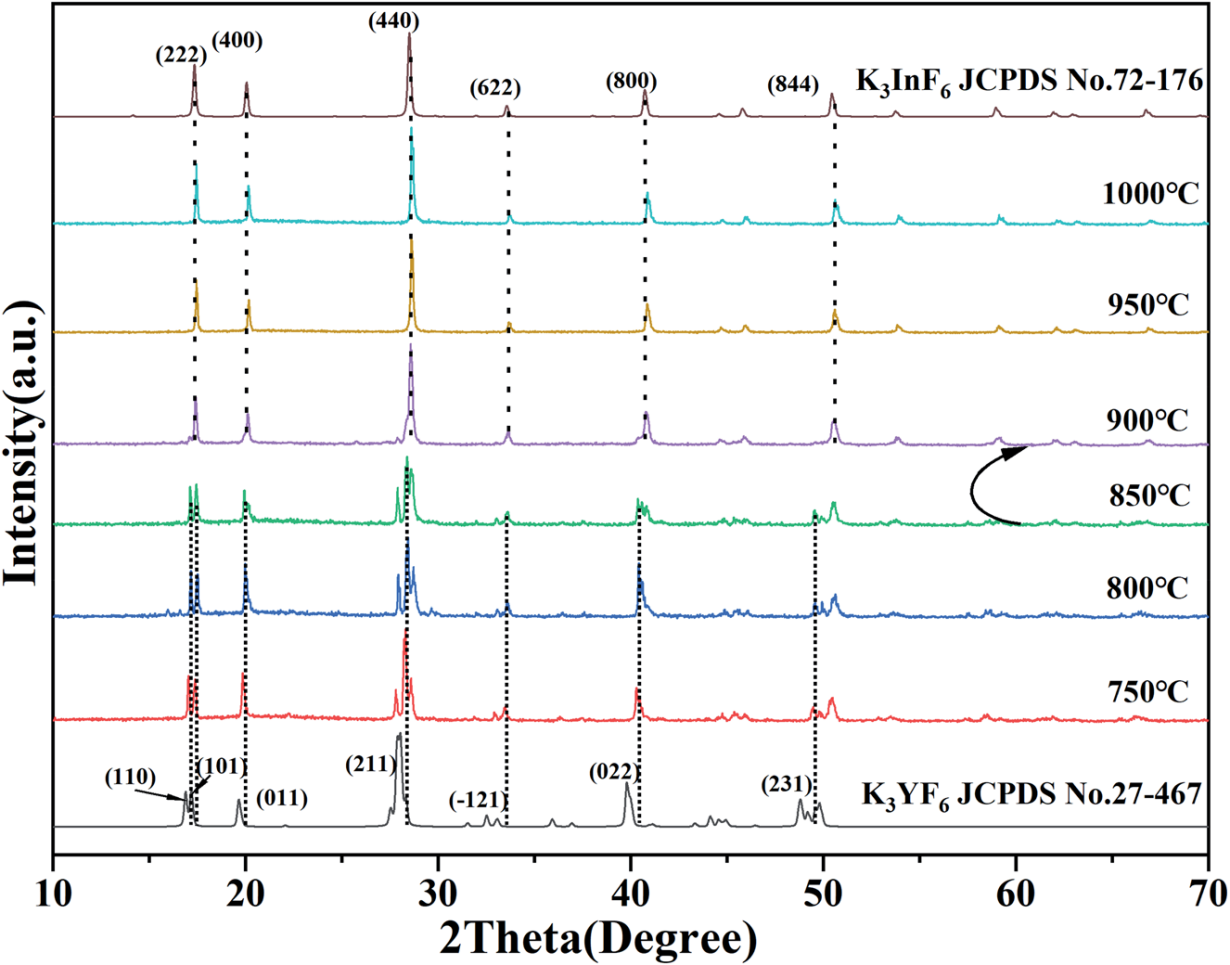
XRD patterns of KSLF at different synthesis temperatures.

Fig. 2 shows the XRD patterns of K_3_Sc_0.5_Lu_0.5_F_6_ (KSLF) at different synthesis temperatures. The results showed when the temperature was below 850 °C, the main strong diffraction peaks of KSLF were consistent with the standard card of the monoclinic K_3_YF_6_ (JCPDF no. 27-467). Meanwhile, when the synthesis temperature was above 900 °C, the diffraction peaks of KSLF were consistent with the standard card of cubic K_3_InF_6_ (JCPDS no. 72-176). It shows that as the synthesis temperature increases, the crystal structure of the sample changes from monoclinic to cubic and all diffraction peaks become sharper which indicates that the crystallinity becomes better.^34,37^

**Fig. 3:**
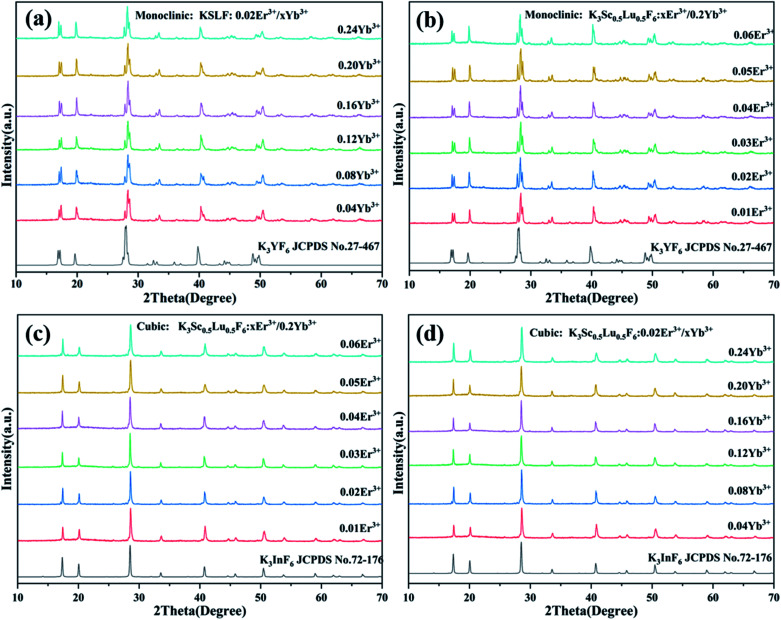
(a and c) XRD patterns of the monoclinic and cubic KSLF: 0.02Er^3+^, *x*Yb^3+^(*x* = 0.04, 0.08, 0.12, 0.16, 0.20 and 0.24), the standard pattern of K_3_YF_6_ (JCPDF no. 27-467) and K_3_InF_6_ (JCPDS no. 72-176) are shown as references; (b and d) XRD patterns of the monoclinic and cubic KSLF: *x*Er^3+^, 0.2Yb^3+^ (*x* = 0.01, 0.02, 0.03, 0.04, 0.05 and 0.06), the standard patterns of K_3_YF_6_ (JCPDF no. 27-467) and K_3_InF_6_ (JCPDS no. 72-176) are shown as references.

The luminous efficiency of up-conversion luminescent materials is not only related to the host structure, but also related to the phase purity of the prepared materials. Fig. 3(a and b) shows the XRD patterns of the monoclinic KSLF: 0.02Er^3+^, *x*Yb^3+^ and KSLF: *x*Er^3+^, 0.2Yb^3+^, and the data of monoclinic K_3_YF_6_ (JCPDF no. 27-467) is shown as a reference. It is clear that the XRD diffraction peaks of KSLF: 0.02Er^3+^, *x*Yb^3+^ (*x* = 0.04, 0.08, 0.12, 0.16, 0.20 and 0.24) and KSLF: *x*Er^3+^, 0.2Yb^3+^ (*x* = 0.01, 0.02, 0.03, 0.04, 0.05 and 0.06) match well with the standard card of K_3_YF_6_ (JCPDF no.27-467), indicating all the samples belong to the monoclinic form with a space group of *P*2_1_/*n*. Fig. 3(c and d) shows the XRD patterns of the cubic KSLF: 0.02Er^3+^, *x*Yb^3+^ and KSLF: *x*Er^3+^, 0.2Yb^3+^, and the with cubic K_3_InF_6_ (JCPDS no. 72-176) as a standard. According to the patterns, we can learned: XRD diffraction peaks of cubic KSLF: 0.02Er^3+^, *x*Yb^3+^ (*x* = 0.04, 0.08, 0.12, 0.16, 0.20 and 0.24) and KSLF: *x*Er^3+^, 0.2Yb^3+^ (*x* = 0.01, 0.02, 0.03, 0.04, 0.05 and 0.06) match well with the standard card of K_3_YF_6_ (JCPDF no.27-467). It shows that the synthesized samples belong to the cubic system, with a space group of *Fd*3̄.^38–40^ The samples for the two different crystal structures are all pure phases. The introduction of Er^3+^, Yb^3+^ did not have any significant influence on the crystal form of KSLF.^29^

**Fig. 4:**
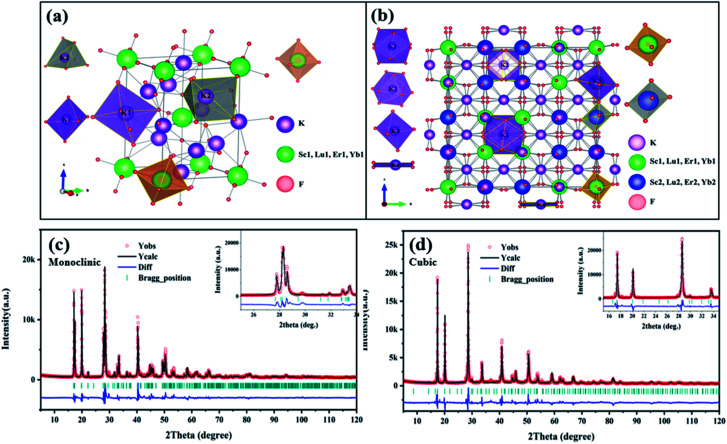
(a and b) The crystallographic structure of monoclinic and cubic KSLF: 0.04Er^3+^, 0.2Yb^3+^; (c and d) Rietveld refinement XRD patterns of monoclinic and cubic KSLF: 0.04Er^3+^, 0.2Yb^3+^.

The crystal structure of monoclinic and cubic KSLF: 0.04Er^3+^, 0.2Yb^3+^, and coordination environments of K, Sc, Lu, Er, Yb and F are presented. Fig. 4(a) is the crystal structure of monoclinic KSLF, the space group is *P*2_1_/*n*, Sc, Lu, Er and Yb coordinate to six F to form [Sc, Lu, Er, YbF_6_] regular octahedra. There are two non-equivalent positions of K in the crystal structure of KSLF: 0.04Er^3+^, 0.2Yb^3+^, one with twelve-fold coordination and another with six-fold coordination.^41^Fig. 4(b) is the crystal structure of cubic KSLF: 0.04Er^3+^, 0.2Yb^3+^, the space group is *Fd*3̄, K occupies four different crystallographic sites named K (1), K (2), K (3) and K (4), respectively. Sc1, Lu1, Er1 and Yb1 are situated in the center of the regular octahedron with 6-fold coordination by F^−^, Sc2, Lu2, Er2 and Yb2 are situated in the center of the deformed octahedron with 6-fold coordination by F^−^.^42^

Fig. 4 shows the Rietveld refinement of monoclinic and cubic KSLF: 0.04 Er^3+^, 0.2Yb^3+^, where the red circles, black solid line, short green vertical and blue solid lines represent the observed pattern obtained from XRD measurements, the calculated pattern, the Bragg positions, and the difference between the observed and calculated patterns, respectively. In Fig. 4(c), all peaks were indexed by monoclinic crystal with parameters close to those of previously reported K_3_InF_6_ compound, and the structural parameters of K_3_InF_6_ were used as initial parameters in the Rietveld analysis. In Fig. 4(d), all peaks were indexed by cubic crystal with parameters close to those of previously reported K_3_YF_6_ compound, and the structural parameters of K_3_YF_6_ were used as initial parameters in the Rietveld analysis. Sites of Sc/Lu ions in monoclinic and cubic KSLF: 0.04 Er^3+^/0.2Yb^3+^ are occupied by Er, Yb ions. The final refinement is stable and convergent well with low residual factors *R*_p_ = 7.804%, *χ*^2^ = 3.032 and *R*_p_ = 9.331%, *χ*^2^ = 3.452, indicating no unidentified diffraction peaks from impurity. The final refined crystallographic data are listed in Table 1. The cell parameters of monoclinic KSLF: 0.04 Er^3+^, 0.2Yb^3+^ are determined to be *a* = 6.257 Å, *b* = 6.439 Å, *c* = 8.930 Å and *V* = 359.79 Å^3^, and the cell parameters of cubic KSLF: 0.04 Er^3+^, 0.2Yb^3+^ are determined to be *a* = 17.707 Å and *V* = 5551.4 Å^3^. The crystallographic site coordinates, occupancy factors, and equivalent isotropic displacement parameters of monoclinic and cubic KSLF: 0.04 Er^3+^, 0.2Yb^3+^ are summarized in Tables 2 and 3.^43,44^ Based on the site occupation fraction in Rietveld refinement, the Er^3+^ ions take up ∼5% Sc/Lu sites and Yb^3+^ ions take up ∼24.9% Sc/Lu sites in monoclinic KSLF. In cubic KSLF, the Er1^3+^ ions take up ∼2.7% Sc1/Lu1 sites and Yb1^3+^ ions take up ∼20.2% Sc1/Lu1 sites, the Er2^3+^ ions take up ∼3.6% Sc2/Lu2 sites and Yb2^3+^ ions take up ∼20.3% Sc2/Lu2 sites.^45,46^

**Table 1:** Refined structural parameters for monoclinic and cubic KSLF: 0.04Er^3+^, 0.2Yb^3+^ sample obtained from the Rietveld refinement using X-ray powder diffraction data

Compound	Monoclinic	Cubic
2*θ*	5–120	5–120
Symmetry	Monoclinic	Cubic
Space group	*P*2_1_/*n*	*Fd*3̄
*a*/Å	6.257 (2)	17.707 (1)
*b*/Å	6.439 (1)	
*c*/Å	8.930 (2)	
*β* (degree)	90.304 (2)	
Volume/Å^3^	359.79 (3)	5551.4 (1)
*Z*	2	32
*R*_p_ (%)	7.804%	9.331%
*R*_wp_ (%)	11.194%	12.670%
*χ* ^2^	3.032	3.452

**Table 2:** Fractional atomic coordinates and occupancy parameters of monoclinic KSLF: 0.04Er^3+^, 0.2Yb^3+^

Atom	Mult.	*x*	*y*	*z*	Occ.	Biso
Sc1	0	0	0	0	0.320(4)	0.50(14)
Lu1	0	0	0	0	0.425(7)	0.50(14)
Yb1	0	0	0	0	0.249(15)	0.50(14)
Er1	0	0	0	0	0.05(2)	0.50(14)
K1	0	0.5	0.5	0	1	1.00(28)
K2	0	0.0111(11)	0.55233(60)	0.74408(58)	1	1.00(26)
F1	0	0.2163(18)	0.3223(18)	0.5250(16)	1	1.00(44)
F2	0	0.3648(21)	0.7871(19)	0.5504(13)	1	1.00(47)
F3	0	0.4273(17)	0.5318(15)	0.2775(13)	1	0.60(40)

**Table 3:** Fractional atomic coordinates and occupancy parameters of cubic KSLF: 0.04Er^3+^, 0.2Yb^3+^

Atom	Mult.	*x*	*y*	*z*	Occ.	Biso
Sc1	16	0	0	0	0.326	0.17(46)
Sc2	16	0.5	0.5	0.5	0.231	0.10(43)
Lu1	16	0	0	0	0.445	0.17(46)
Lu2	16	0.5	0.5	0.5	0.5304	0.10(43)
Yb1	16	0	0	0	0.202(12)	0.17(46)
Yb2	16	0.5	0.5	0.51	0.203(11)	0.10(43)
Er1	16	0	0	0	0.027	0.17(46)
Er2	16	0.5	0.5	0.5	0.036	0.10(43)
K1	8	0.125	0.125	0.125	1	1.0(23)
K2	8	0.625	0.625	0.625	1	1.0(24)
K3	32	0.25	0.25	0.25	1	1.00(27)
K4	48	0.375	0.125	0.125	1	1.00(65)
F1	96	0.1112(14)	0.0140(15)	0.9626(10)	1	1.00(64)
F2	96	0.6131(12)	0.4981(13)	0.5069(10)	1	1.00(44)

**Fig. 5:**
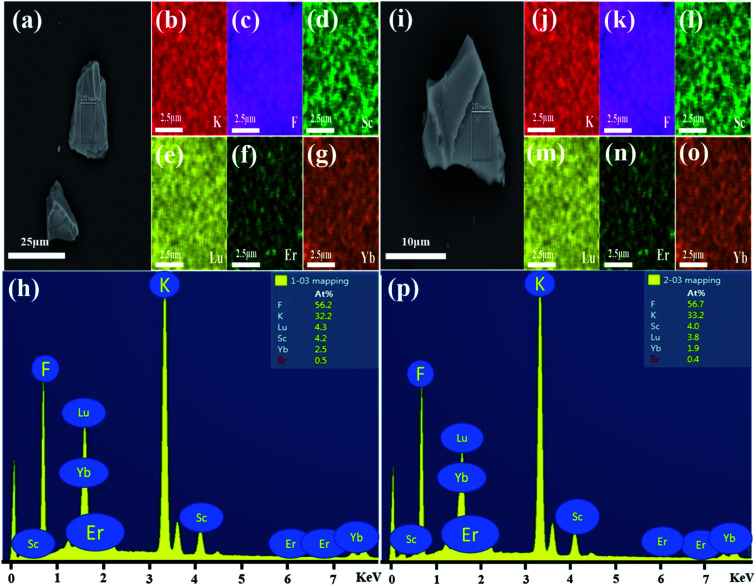
(a) SEM image of monoclinic KSLF: 0.04Er^3+^, 0.2Yb^3+^; (b–g) monoclinic KSLF: 0.04Er^3+^, 0.2Yb^3+^ sample element mapping; (h) EDX spectrum of monoclinic KSLF: 0.04Er^3+^/0.2Yb^3+^ sample; (i) SEM image of cubic KSLF: 0.04Er^3+^, 0.2Yb^3+^; (j–o) cubic KSLF: 0.04Er^3+^, 0.2Yb^3+^ sample element mapping; (p) EDX spectrum of cubic KSLF: 0.04Er^3+^, 0.2Yb^3+^ sample.

Fig. 5(a) shows the SEM image of monoclinic KSLF: 0.04Er^3+^, 0.2Yb^3+^, the prepared sample is irregularly granular, with a particle size of about tens of microns. In order to understand the distribution of all elements in the sample, a square is selected as the area for element mapping and EDS testing. Fig. 5(b–g) shows the element mapping images, it can be seen that K, Sc, Lu, F, Er can be observed in monoclinic KSLF: 0.04Er^3+^, 0.2Yb^3+^ and all the elements in the sample are homogenously distributed over the granules. Fig. 5(h) depicts the EDX spectrum and the atomic composition ratios of monoclinic KSLF: 0.04 Er^3+^, 0.2Yb^3+^ sample. Fig. 5(i) shows the SEM image of cubic KSLF: 0.04Er^3+^, 0.2Yb^3+^, choose a square as the area for element mapping and EDS testing. According to the mapping results of Fig. 5(j–o), all elements are evenly distributed, and the existence of Er illustrates that Er^3+^ ions were successfully doped into the crystal lattice. Fig. 5(p) shows the EDX spectrum and the atomic composition ratios of cubic KSLF: 0.04 Er^3+^, 0.2Yb^3+^ sample. For both monoclinic and cubic KSLF: 0.04Er^3+^, 0.2Yb^3+^, the molar ratio of Sc to Lu is close to 1 : 1, and the actual doping amount of Er and Yb is also close to the theoretical doping amount, which further shows that the measured atomic ratio of the corresponding element is close to the calculated value.

**Fig. 6:**
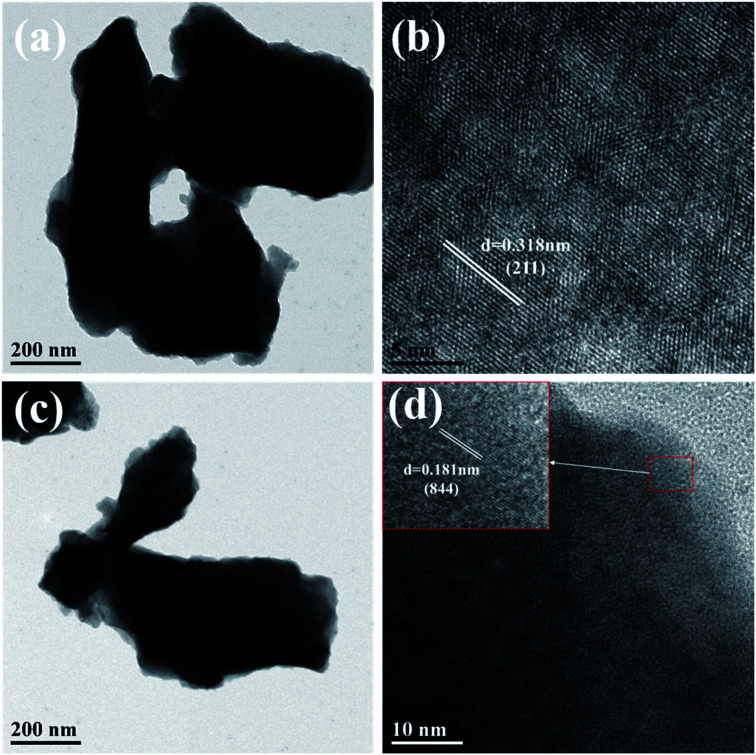
(a and b) are TEM and HR-TEM images of monoclinic KSLF: 0.04Er^3+^, 0.2Yb^3+^; (c and d) are TEM and HR-TEM images of cubic KSLF: 0.04Er^3+^, 0.2Yb^3+^.

The microstructures of monoclinic and cubic KSLF: 0.04Er^3+^, 0.2Yb^3+^ were further characterized by TEM. In Fig. 6, the enlarged HRTEM image showed the characteristic lattice fringe of monoclinic and cubic KSLF: 0.04Er^3+^, 0.2Yb^3+^. The monoclinic KSLF: 0.04Er^3+^, 0.2Yb^3+^ has a lattice plane spacing of 0.318 nm, and the corresponding lattice plane index is (211). The cubic KSLF: 0.04Er^3+^, 0.2Yb^3+^ has a lattice plane spacing of 0.181 nm, and the corresponding lattice plane index is (844).

**Fig. 7:**
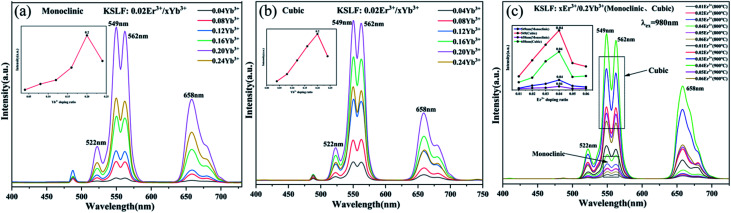
(a and b) UC spectra (*λ*_ex_ = 980 nm) of monoclinic and cubic KSLF: 0.02Er^3+^, *x*Yb^3+^: *x* = 0.02, *y* = 0.04, 0.08, 0.12, 0.16, 0.20 and 0.24); (c) UC spectra (*λ*_ex_ = 980 nm) of monoclinic and cubic KSLF: 0.02Er^3+^, *x*Yb^3+^: *x* = 0.02, *y*=(0.04, 0.08, 0.12, 0.16, 0.20 and 0.24); the insets depict the relative emission-intensity trends for the 549 nm peak in terms of the Er^3+^ concentration.

Fig. 7(a and b) shows the up-conversion emission spectra of monoclinic and cubic KSLF: 0.02Er^3+^, *x*Yb^3+^(*x* = 0.04, 0.08, 0.12, 0.16, 0.20 and 0.24) for different Yb^3+^ doping ratio. As Yb^3+^ concentration changes from 0.04 to 0.24 mol, the emission intensity of the sample first increases and then decreases. When the doping ratio of Yb^3+^ is 0.20 mol, the emission intensity centered at 549 nm reaches the maximum. Fig. 7(c) shows the up-conversion luminescence performance of cubic and monoclinic KSLF: *x*Er^3+^, 0.2Yb^3+^ (*x* = 0.01, 0.02, 0.03, 0.04, 0.05 and 0.06) under 980 nm excitation. Er^3+^, Yb^3+^ co-doped KSLF showed bright green emission at 549 nm, and a weak red emission peak appeared at 657 nm. Keeping the doping ratio of Yb^3+^ at 0.2, it can be clearly seen that in monoclinic and cubic forms, as Er^3+^ increases from 0.01 to 0.06, the emitted up-conversion luminous intensity at 549 nm first increases and then shows a downward trend. When the Er^3+^ doping ratio is 0.04, the up-conversion luminescence intensity reaches the maximum value, and then the concentration quenching effect appears. This is because as Er^3+^ concentration increases, the central ion distance decreases to be less than the critical distance. In the process of energy transfer, the possibility of energy transfer in quenching center increases.^47^ The energy is released from the quenching center, resulting in the decrease of up-conversion luminescence intensity. This can be visually illustrated by the trend graph of the peak intensity at 549 nm and 658 nm with the concentration of Er^3+^ for monoclinic and cubic form. It can be observed that crystal form has no effect on the position of spectral peaks, but the luminescence intensity of cubic form is significantly higher than that of monoclinic form. This is because in monoclinic and cubic system, the crystal field environments are different, which affects the up-conversion energy transfer process, especially the non-radiation transition process of Er^3+^. The probability of the non-radiation transition of Er^3+^ in monoclinic crystal field environment is greater than that in cubic system, which leads to the stronger luminescent intensity of cubic form.

**Fig. 8:**
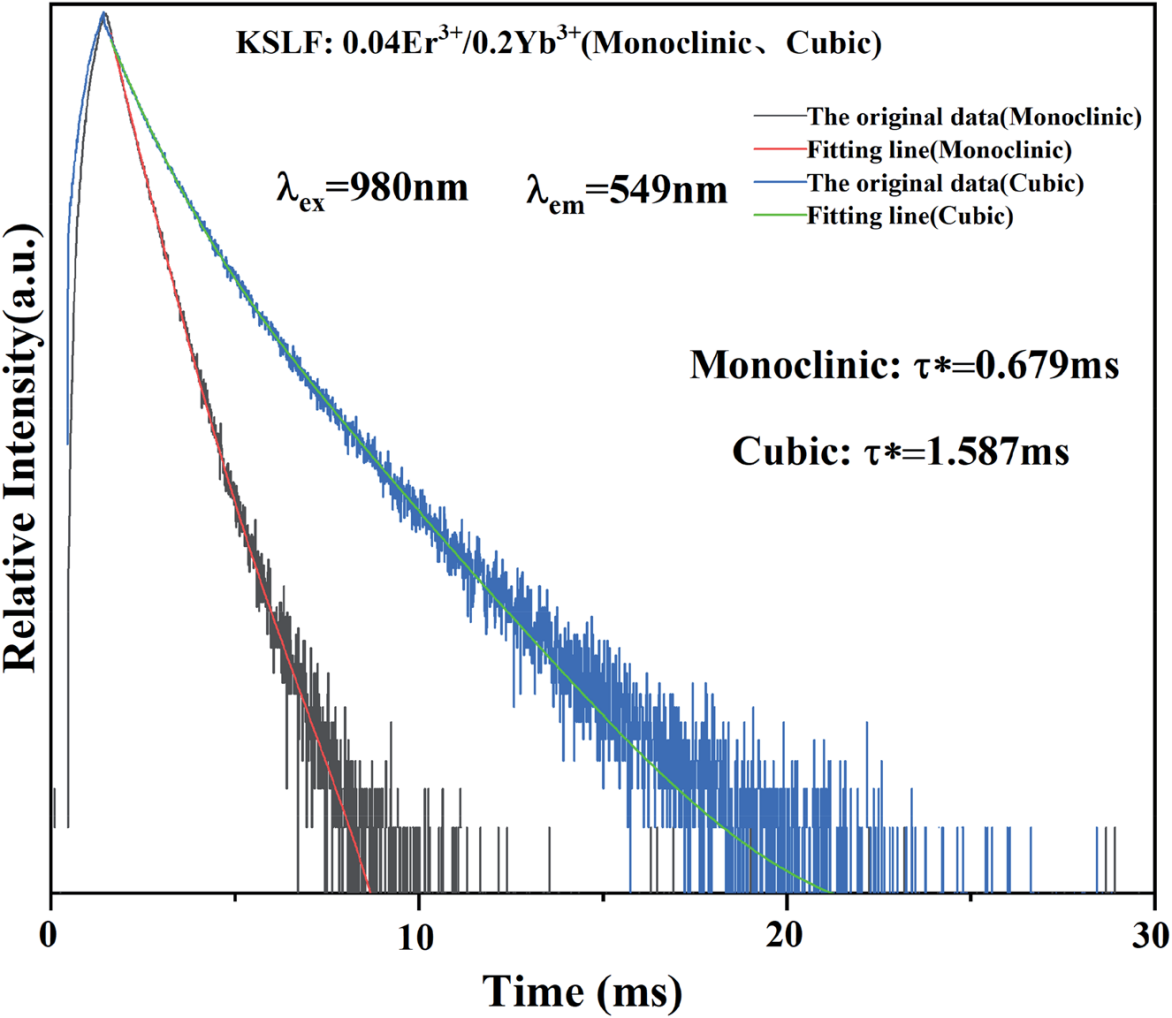
Fluorescence decay curve of monoclinic and cubic KSLF: 0.04Er^3+^, 0.2Yb^3+^ under the excitation of 980 nm.

The photoluminescence decay curves of the prepared monoclinic and cubic KSLF: 0.04Er^3+^, 0.2Yb^3+^ are shown in Fig. 8. The attenuation curve is fitted with a double exponential eqn (1):

1 *I*(*t*) = *I*_0_ + *A*_1_ exp(−*t*/*τ*_1_) + *A*_2_ exp(−*t*/*τ*_2_)

Among them, *I*(*t*) and *I*_0_ are the luminous intensity and background intensity at time *t*, respectively, *A*_1_ and *A*_2_ are the emission intensity factors, and *τ*_1_ and *τ*_2_ are the decay time of the exponential component, respectively.^48^ The average life span can be calculated as follows:

2 *τ*_ave_ = (*A*_1_*τ*_1_^2^ + *A*_2_*τ*_2_^2^)/(*A*_1_*τ*_2_ + *A*_2_*τ*_1_)

Based on the equations, the calculated average lifetimes are about 0.679 ms and 1.587 ms for monoclinic and cubic KSLF: 0.04Er^3+^, 0.2Yb^3+^ phosphors. For samples with monoclinic form, the fluorescence lifetimes are comparatively shorter (0.679 ms), and the lifetimes for samples with the cubic form are notably longer (1.587 ms).

**Fig. 9:**
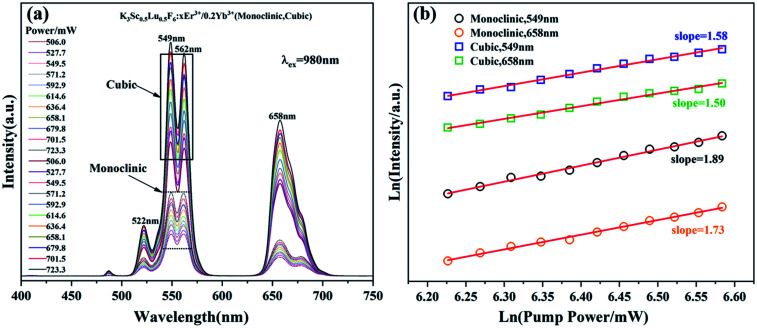
(a) Pump power up-conversion spectra of monoclinic and cubic KSLF: 0.04Er^3+^, 0.2Yb^3+^; (b) the relationship between green and red up-conversion luminous intensity and pump power at 549 nm and 658 nm for monoclinic and cubic KSLF: 0.04Er^3+^, 0.2Yb^3+^.

The up-conversion emission spectra of monoclinic and cubic KSLF: 0.04Er^3+^, 0.2Yb^3+^ samples under different pump powers are shown in Fig. 9. In the excitation power range is 506.0–723.3 mW, the influence of the pump power on the up-conversion emission intensity is studied. It can be seen from Fig. 9(a) that the Er^3+^ in the two samples with different crystal structures has obvious green and red emission at 549 nm and 658 nm, and the emission intensity shows an obvious linear upward trend. According to the relationship between the up-conversion luminous intensity (*I*) and the excitation power (*P*), *I* ∝ *P*_*n*_, where *n* is the number of photons required from the ground state to the emission state during the up-conversion period.^22^ The integrated emission intensity of green and red light of KSLF: 0.04Er^3+^, 0.2Yb^3+^ at 549 nm and 658 nm is plotted with different pump powers in the form of Ln–Ln (pump power–emission intensity) curves. For monoclinic KSLF: 0.04Er^3+^, 0.2Yb^3+^, the slopes of green light and red light are 1.89 and 1.73 respectively, indicating that both green light (549 nm) and red light (658 nm) are two-photon processes produced. For cubic KSLF: 0.04Er^3+^, 0.2Yb^3+^, the slopes of green light and red light are 1.58 and 1.50, respectively, indicating that green light (549 nm) and red light (658 nm) are also two-photon processes produced.

**Fig. 10:**
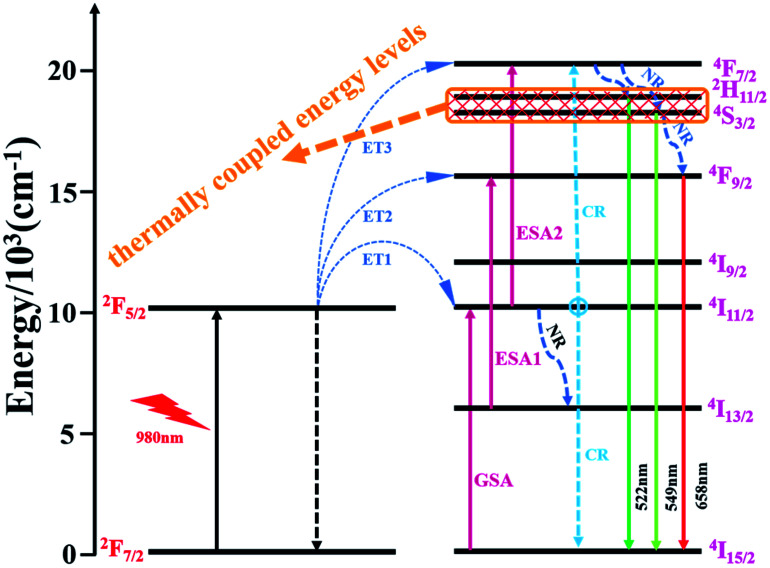
Schematic energy levels diagram of Er^3+^ and Yb^3+^ system.

In order to illustrate the luminescence mechanism of KSLF: 0.04Er^3+^, 0.2Yb^3+^, the possible UC processes and energy levels diagram are described in detail in Fig. 10. The main UC processes include energy transfer (ET), ground state absorption (GSA), excited state absorption (ESA) and cross relaxation (CR). In the energy levels diagram, the energy gap of Er^3+^ (^4^I_15/2_ and ^4^I_11/2_) is match well with the energy gap of Yb^3+^ (^2^F_7/2_ and ^2^F_5/2_), so the ET from Yb^3+^ to Er^3+^ may occur: ET1: Er^3+^(^4^I_15/2_) + Yb^3+^(^2^F_5/2_) → Er^3+^(^4^I_11/2_) + Yb^3+^(^2^F_7/2_). For 522 nm, 549 nm and 562 nm emission peaked of the Er^3+^, ^4^F_7/2_ can populate the excited energy levels ^2^H_11/2_ and ^4^S_3/2_ by non-radiative (NR). And the ^4^F_7/2_ states could follow three processes: excitation states absorption (ESA2), ET3 and cross relaxation (CR). For red emitting centered at 658 nm from Er^3+^, the population of ^4^F_9/2_ level involves three processes: ESA1, ET2 and NR.

**Fig. 11:**
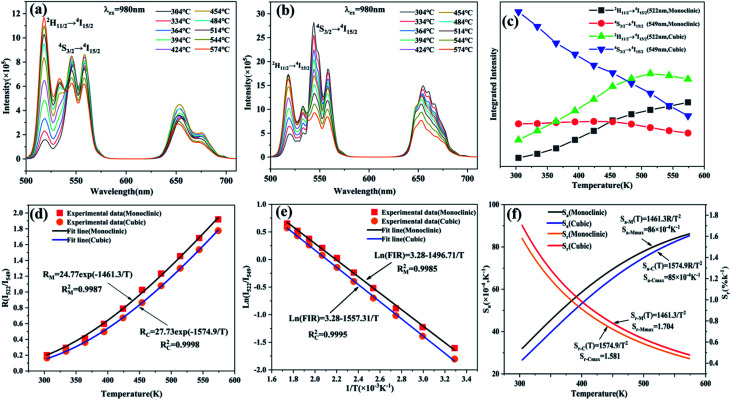
(a and b) The temperature-dependent UC luminescence behavior of monoclinic and cubic KSLF; (c) the temperature-dependent integrated UCL intensity map of the emission peaks of monoclinic and cubic KSLF: 0.04 Er^3+^, 0.20Yb^3+^ at 522 nm and 549 nm; (d) dependence of the FIR of I_525_/I_549_ on temperature and the fitting curve; (e) variations of FIR (I_522/549_) for monoclinic and cubic KSLF: 0.04 Er^3+^, 0.20Yb^3+^ as a function of temperature and corresponding linear fitting; (f) calculated sensitivities (*S*) of KSLF: 0.04 Er^3+^, 0.20Yb^3+^ with the assistance of fitting equation in (e).

Depending on the excellent up-conversion luminescence performance of KSLF: Er^3+^, Yb^3+^, which can crystallize into two crystal forms, the temperature sensing performance has been investigated. Fig. 11(a) and (b) shows the green UC emission spectra of monoclinic KSLF: Er^3+^, Yb^3+^ and cubic KSLF: Er^3+^, Yb^3+^ under 980 nm excitation at 304–574 K. The spectra show systematic changes as the temperature increased from 304 to 574 K. With the increase of temperature, the intensity at 549 nm of the two crystal forms of KSLF: Er^3+^, Yb^3+^ decreased, while the intensity at 522 nm increased significantly (Fig. 11 c). Temperature sensing mainly uses two emission lines, and the energy gap between the two is small. As the temperature increases, the higher energy levels become denser due to the thermalization of the lower energy levels. Because the energy gap between ^2^H_11/2_ and ^4^S_3/2_ is small, the ^2^H_11/2_ state could be populated from ^4^S_3/2_ by thermal excitation, which leads to the variations of emission intensity of ^2^H_11/2_ and ^4^S_3/2_ transitions at elevated temperature.^49^ The relative population of the “thermally coupled” ^2^H_11/2_ and ^4^S_3/2_ levels is a quasithermal equilibrium obeying Boltzmann-type distribution, because the emission intensity varies as a function of temperature.^50^ Potential temperature measurement applications are related to fluorescence intensity ratio (FIR), which can be evaluated using the following formula:3
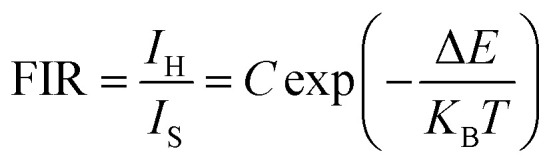
where *I*_H_ and *I*_S_ are the emission fluorescence intensity of the high thermal coupling level (^2^H_11/2_) and low thermal coupling level (^4^S_3/2_), *C* is the temperature-independent constant, and Δ*E* represents the energy gap between ^2^H_11/2_ and 4S_3/2_. *K*_B_ = 0.695 K^−1^ cm^−1^ is Boltzmann's constant and *T* is absolute temperature. According to the formula, the scatter plot of the experimental data can be fitted well to obtain the function expression. As shown in Fig. 11(d), in the temperature range of 304–574 K, the FIR of the 522 nm and 549 nm bands changes with temperature, the monoclinic form can be determined as *R*_M_ = 24.77 exp(−1461.3/*T*), and the cubic form can be determined as *R*_C_ = 27.73 exp(−1574.9/*T*). It can be clearly seen that FIR increases significantly with temperature.^51^ Here, the emission intensity at 522 nm and 549 nm is used to evaluate the ^2^H_11/2_ and ^4^S_3/2_ transitions for simplification. Eqn (4) can be derived from eqn (3) as follow:4
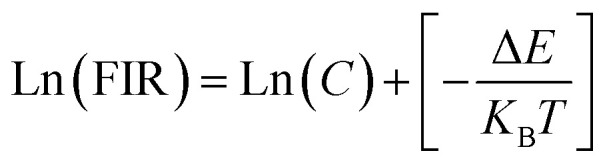


Fig. 11(e) shows the relationship between Ln(FIR) and 1/*T* of monoclinic and cubic KSLF: 0.04Er^3+^, 0.2Yb^3+^ sample in the temperature range of 304–574 K. The monoclinic data can be fitted as: Ln(FIR) = 3.28–1496.71/*T*, the slope −Δ*E*/*k* = −1123.6 and the intercept Ln(*C*) = 3.28, Δ*E* = 1040 cm^−1^. The cubic data can be well fitted as: Ln(FIR) = 3.28–1557.31/*T*, the slope −Δ*E*/*k* = −1557.31 and the intercept Ln(*C*) = 3.28, Δ*E* = 1082 cm^−1^. It is close to the actual energy gap between ^4^S_3/2_ and ^2^H_11/2_ levels in the current case. In order to evaluate the actual sensing ability of sensor materials, absolute sensitivity (*S*_a_) and relative sensitivity (*S*_r_) are usually used to study the sensitivity change with temperature, which is defined as follows:^52^5
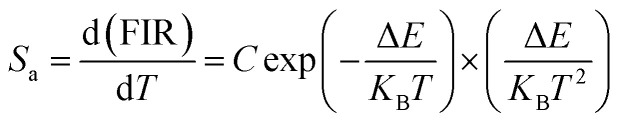
6
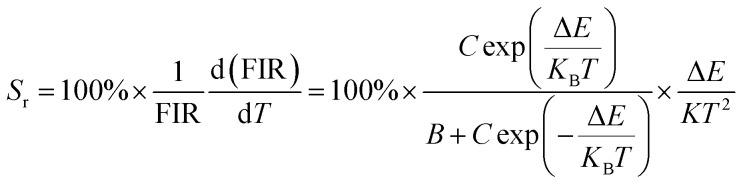


Fig. 11(f) is the calculated absolute temperature sensitivity and relative temperature sensitivity, and their fitting curves with temperature from 304 K to 574 K. With the increase of temperature from 304 K to 574 K, *S*_a_ displays a monotonous downward trend and *S*_r_ displays a monotonous upward trend for both monoclinic and cubic forms. For monoclinic form, the maximum value of *S*_a_ reaches 86 × 10^−4^ K^−1^, while the maximum relative sensitivity *S*_r_ is 1.704% K^−1^. For cubic form, the maximum value of *S*_a_ reaches 85 × 10^−4^ K^−1^, while the maximum relative sensitivity *S*_r_ is 1.581% K^−1^. The *S*_a_ in monoclinic and cubic forms is not much different, and the *S*_r_ in monoclinic form is greater than that in cubic form. Several classics optical thermometers are listed in Table 4. The results demonstrate that the monoclinic and cubic KSLF: Er^3+^, Yb^3+^ are promising in temperature sensing.

**Table 4:** The absolute sensitivity of different Er^3+^/Yb^3+^ doped matrices and the temperature range

RE doped samples	Maximum absolute sensitivity (×10^−4^ K^−1^)	Temperature range (K)	Ref.
CaMoO_4_: Er^3+^, Yb^3+^	72	300–760	53
KSLF: Er^3+^, Yb^3+^ (monoclinic)	86	304–574	This work
KSLF: Er^3+^, Yb^3+^ (cubic)	85	304–574	This work
NaGdTiO_4_: Er^3+^, Yb^3+^	45	300–510	54
Gd_2_MoO_4_: Er^3+^, Yb^3+^	53	303–703	14
Na_0.5_Bi_0.5_TiO_3_: Er^3+^, Yb^3+^	35	173–553	55
Y_2_O_3_: Er^3+^, Yb^3+^	97	314–573	1
La_2_O_2_S: Er^3+^, Yb^3+^	80	290–573	56
CaLa_2_ZnO_5_: Er^3+^, Yb^3+^	59	298–513	57
LuVO_4_: Er^3+^, Yb^3+^	67	100–500	2
Ba_3_Y_4_O_9_: Er^3+^, Yb^3+^	45.8	298–573	13
NaY(WO_4_)_2_: Er^3+^, Yb^3+^	61	30–300	58
Ba_5_Gd_8_Zn_4_O_21_: Er^3+^, Yb^3+^	24	298–573	59
Na_2_Gd_2_Ti_3_O_10_: Er^3+^, Yb^3+^	58	290–490	60
YVO_4_: Er^3+^, Yb^3+^	117	300–485	61

## 4. Conclusions

In summary, monoclinic and cubic KSLF: Er^3+^, Yb^3+^ were prepared by high temperature solid state method, a combination of XRD, SEM, TEM, XPS shows that KSLF: Er^3+^, Yb^3+^ with monoclinic (*P*2_1_/*n*) and cubic (*Fd*3̄) systems can be mutual transformed at different temperatures. Up-conversion fluorescence spectrum shows that the luminescence intensity of the cubic form is significantly higher than that of the monoclinic form, that may be because the probability of the non-radiation transition of Er^3+^ in the monoclinic crystal field environment is greater than that in the cubic system. The average lifetimes are about 0.679 ms and 1.587 ms for monoclinic and cubic KSLF: 0.04Er^3+^, 0.2Yb^3+^ phosphors. According to the fitting result of pump power dependence of up-conversion intensity, the green and red up-conversion emission of monoclinic and cubic KSLF: Er^3+^, Yb^3+^ all belong to the two-photon process. For the monoclinic and cubic forms, the maximum value of *S*_a_ reaches 86 × 10^−4^ K^−1^ and 85 × 10^−4^ K^−1^, indicating that the as-prepared KSLF: Er^3+^, Yb^3+^ phosphor is appropriate for practical application in optical temperature sensors.

## Conflicts of interest

There are no conflicts to declare.

## Supplementary Material
